# 

**Published:** 1896-07

**Authors:** 


### CONTENTS:

### SELECTIONS.

Article I.—Anæsthesia, Local and General. By A. C. Hewitt, M. D. - - - - - - - - - 97
" II.—Notes Upon Dentine and Enamel. By Charles S. Tomes, M. A., F. R. S. - - - - - 106
" III.—Air-Chambers. Dr. L. P. Haskell. - - - 118
" IV.—Cataphoresis for Obtunding Sensitive Dentin. By Dr. H. L. Ambler. - - - - - - 120
" V.—Duration of Pulpless Teeth. By Dr. Taft. - - 124
" VI.—A Case of Removal of the Major Portion of the Lower Jaw for Sarcoma. By Thomas S. K. Morton, M. D. - - - - - - - - - - 126
" VII.—Acute Abscess of Teeth. By Dr. M. L. Rhein. - 129
" VIII.—Treatment of Abscessed Teeth. By Dr. M. L. Rhein. 132

### EDITORIAL, ETC.

University of Buffalo, Dental Department. - - - - 134
Test for Insane Persons. - - - - - - - - - 134

### OBITUARY.

James Taylor, M. D., D. D. S. - - - - - - - 135

### MONTHLY SUMMARY.

Cohesion and Adhesion. - - - - - - - - 137

Tooth in Her Lung. - - - - - - - - - 138

Repairing Broken Down Teeth. - - - - - - - 139

The Advertising Dentist. - - - - - - - - 140

What We Need. - - - - - - - - - 140

Dental Hemorrhage. - - - - - - - - - 141

Carbolic Acid. - - - - - - - - - 141

No Toothache in that Factory. - - - - - - - 142

Insomnia. - - - - - - - - - - 142

Chronic Gastritis of Long Standing, with Periodic Attacks of Migraine. - - - - - - - - - 143

Calcareous Deposits During Pregnancy. - - - - - 144

Bad Breath. - - - - - - - - - - 144

